# Growing through adversity: the relation of early childhood educator post-traumatic growth to young children’s executive function

**DOI:** 10.3389/fpsyg.2024.1272294

**Published:** 2024-03-13

**Authors:** Caron A. C. Clark, Holly Hatton-Bowers, Kimia Akhavein, Sarah Rasby, Gilbert R. Parra

**Affiliations:** ^1^Department of Educational Psychology, University of Nebraska-Lincoln, Lincoln, NE, United States; ^2^Department of Child, Youth, and Family Studies, University of Nebraska-Lincoln, Lincoln, NE, United States; ^3^Department of Psychology, University of Nebraska-Lincoln, Lincoln, NE, United States

**Keywords:** early childhood education, adverse childhood experiences, post-traumatic growth, teacher well-being, executive function, preschool, trauma

## Abstract

**Introduction:**

Early childhood educators (ECEs) play a critical role in supporting the development of young children’s executive functions (EF). EF, in turn, underpins lifelong resilience and well-being. Unfortunately, many ECEs report adverse childhood experiences (ACEs) that may compound high stress levels associated with an emotionally and physically demanding profession. ACEs have well-established negative implications for adult well-being and may dampen ECEs’ capacities to engage in emotionally responsive interactions with children. However, many individuals who experience ACEs also report post-traumatic growth experiences that foster empathy, self-determination, and resilience. Such post-traumatic growth may equip teachers with skills to engage in responsive interactions with children that support children’s EF. The aim of this study was to explore the relations of ECE ACEs and post-traumatic growth to the EF of children in their classrooms.

**Methods:**

Fifty-three female ECEs self-reported on their ACEs and post-traumatic growth. Parents of 157 children (53% male, 47% female, *M* age = 4.38 years) rated children’s EF.

**Results:**

In a set of linear mixed models that accounted for multiple demographic factors and ECE perceived workplace stressors, ECE ACEs were not significantly related to children’s EF scores. However, controlling for ACEs, higher levels of ECE post-traumatic growth were associated with fewer parent-reported EF difficulties in children.

**Discussion:**

ECEs may draw on the coping skills they have developed in times of adversity to model and promote healthy EF for children. Mental health supports to facilitate ECEs’ processing of their own trauma may be a fruitful means to foster positive early childhood environments that nurture the well-being and resilience of future generations.

## 1 Introduction

Prior to the onset of the Covid-19 pandemic, as many as 63% of 3 to 5 year olds in the USA were enrolled in public or private early childhood education and enrollment rates have rebounded since 2020 (National Center for Education Statistics, 2023). There is growing evidence that high quality early childhood education contributes to children’s long-term academic engagement and achievement, positive relationships, and financial security and healthful behavior in adulthood (Heckman, 2011; Soliday Hong et al., 2019), with these effects being particularly pronounced for children from financially disadvantaged households (van Huizen and Plantenga, 2018). There is also evidence that effects are driven, at least in part, by the positive impact of high quality early education on children’s executive functions (EF), the cognitive processes—including flexible attention, working memory and inhibition—that support children’s self-regulation of their learning, behavior and emotions (Heckman et al., 2012; Blair, 2016; Hatfield et al., 2016). EF is a major predictor of lifelong well-being, academic achievement, and the capacity for resilience in the face of adversity and stress (Masten et al., 2012; Obradović, 2016; Howard et al., 2018; Taylor and Ruiz, 2019; Burchinal et al., 2020). Early childhood settings may therefore provide an especially important social context for fostering children’s long-term resilience through the provision of experiences that support the development of their EF.

Early childhood educators (ECEs) are a lynchpin for providing high quality experiences that nurture children’s healthy development (Hamre, 2014). When educators engage in warm, cognitively stimulating and emotionally responsive interactions with children, children engage in more prosocial peer interactions, exhibit fewer internalizing and externalizing behaviors, and show higher levels of EF, with these benefits persisting as they transition into elementary classrooms (Burchinal et al., 2008; Curby et al., 2009; Cadima et al., 2016; Ragni et al., 2021). The prosocial classroom model proposes that educators set the overall ambience of the classroom and guide children’s learning through their own modulation of their emotions (Jennings and Greenberg, 2009). ECEs with higher levels of emotion regulation and lower psychological distress may be better positioned to engage in responsive, sensitive interactions with children because they are able to attend to children’s emotional cues, respond with equanimity to challenging child behaviors such as conflict or distress, and model effective emotional coping strategies for children (Buettner et al., 2016; Denham and Bassett, 2019). Conversely, educators who have a high burden of psychological distress and/or depleted resources for regulating their emotions may be less able to engage in warm, supportive relationships with children. For instance, they may be preoccupied with their own distress, less able to plan and provide cognitively stimulating experiences for children, more reactive to children’s negative behavior, and less likely to model effective coping and problem-solving strategies (Johnson et al., 2021). Overall, ECEs’ well-being and strategies for emotion regulation theoretically set the stage for their creation of high-quality, positive, responsive experiences that scaffold young children’s EF development.

Unfortunately, many ECEs report high levels of psychosocial distress (Whitebrook et al., 2016; Cassidy et al., 2019) exacerbated by low financial compensation, lack of health benefits, challenging interactions with parents and children, low professional status, high workloads, and physical ill-health (Whitaker et al., 2015; Cumming, 2017; Otten et al., 2019). In addition, many ECEs report clinically significant levels of depression, with some studies indicating rates of depression that are two times higher than in the general population (Whitaker et al., 2013; Kwon et al., 2022). Consistent with the prosocial classroom model, there is some evidence that these high levels of educator stress and depression negatively impact classroom quality, developmentally appropriate teacher beliefs, and teacher-child interactions, as well as relating negatively to children’s socio-emotional adjustment (Li Grining et al., 2010; Jeon L. et al., 2014, 2019; Jeon H.-J. et al., 2019), although effects generally are small and some studies have found that ECE depression does not relate to classroom quality (Roberts et al., 2016; Johnson et al., 2021).

In addition to financial, work-related and psychological stressors, recent studies suggest that many ECEs have experienced adverse or traumatic events in their own childhoods that have well-established, dose-dependent, cumulative negative relations with adult physical and psychological well-being (Dube et al., 2001; Edwards et al., 2003; Petruccelli et al., 2019; Stone et al., 2023). This has inspired calls for more holistic approaches to ECE well-being that consider lifetime adversity experiences and coping resources (Kwon et al., 2022). Adverse Childhood Experiences (ACEs) include physical, emotional, and verbal abuse or neglect, parent mental health difficulties, substance use and incarceration, and parental separation in the first 18 years of life. With each cumulative ACE, the likelihood of depression, post-traumatic stress disorder, anxiety, and psychological distress increases 1.3 to 2-fold (Chang et al., 2019; Petruccelli et al., 2019). Studies also suggest that these traumatic experiences may have inter-generational consequences for children’s development via their impact on caregiver mental health and capacity to engage in warm, sensitive, caregiving responses (Racine et al., 2023; Zhang et al., 2023). For example, maternal ACEs indirectly predict behavior regulation difficulties in children via their relation to maternal avoidant and anxious attachment patterns and depression (Cooke et al., 2019).

In one recent survey of ECEs in the Southeast US, 64% reported at least 1 ACE, and 24% of these educators reported 4 or more ACEs. Moreover, ACEs were moderately associated with workplace emotional exhaustion (Grist and Caudle, 2021). Rates of at least 1 ACE were similar in the Grist and Caudle study to community samples, where around 63% of adults report exposure to 1 ACE, but the number of ECEs reporting 4 or more ACEs was substantially higher than the general community rate of approximately 15% (Ports et al., 2020). In a study of Head Start teachers in Philadelphia (Whitaker et al., 2014), the prevalence of 3 or more ACEs was 23%, and the number of ACE experiences correlated with the degree of ECEs’ current socio-economic hardship, suggesting that educators with the highest financial stress also disproportionately have the highest ACE levels. In another study (Hubel et al., 2020), 73% of ECEs reported at least one ACE, whereas 22% experienced four or more ACEs. A higher number of reported ACEs was linked to lower levels of observed ECE emotional responsiveness toward children in the classroom, showcasing the potential implications of these traumatic early experiences for ECEs’ capacities to provide prosocial classroom environments that facilitate children’s EF (see also Simons et al., 2022; Rancher and Moreland, 2023). Overall, the research suggests that ACEs may take a cumulative toll on ECEs mental health, jeopardizing ECEs capacity to engage in sensitive, responsive interactions that are vital to children’s EF development.

Amidst a wealth of evidence linking ACEs to poor health and mental health outcomes in adulthood (Felitti et al., 1998), there have also been cautions against a deficit-oriented approach and calls to consider positive strategies and resources that may help mitigate the impact of childhood traumatic experiences on well-being (McEwen and Gregerson, 2019). Indeed, many individuals experience or perceive at least some positive change in the face of suffering and adversity (Tedeschi and Calhoun, 2004; Lim and DeSteno, 2016; Greenberg et al., 2018). Post-traumatic growth refers to profound positive transformations in cognitive schemata, psychological attributions and outlook that occur in response to trauma, challenging life events, or adversity (Tedeschi and Calhoun, 2004; Joseph et al., 2012). This growth can include changes in the perception of self, such as an increased sense of competence, self-determination or strength; changes in relationships, such as increased emotional disclosure, connection, and empathy; and changes in philosophy on life, such as an increased sense of meaning and purpose (Tedeschi and Calhoun, 1996). The process of post-traumatic growth involves cognitive re-processing, reflection, and management of distressing emotions. These processes enable individuals to cultivate new perspectives, life narratives, coping resources, and wisdom regarding emotional upheaval (Joseph et al., 2012). Importantly, post-traumatic growth is conceptualized as a protracted response to extreme stress and may occur simultaneously with negative psychological distress (Tedeschi and Calhoun, 1996). While post-traumatic growth and resiliency are not synonymous (Elam and Taku, 2022), they do overlap in the sense that resilience reflects the capacity to grow or flourish in the face of hardship (Duan et al., 2015; Zeidner and Kampler, 2020).

Although there is a dearth of studies examining post-traumatic growth among educators, there is reason to suspect that the new perspectives and coping skills accompanying this growth may play an important role in supporting ECEs’ emotion regulation, psychological well-being, and capacities to support and scaffold children’s EF. Specifically, post-traumatic growth has been linked to enhanced empathy, emotion recognition and emotional reappraisal (Huang et al., 2019; Carter et al., 2021; Elam and Taku, 2022), enhanced use of happiness-increasing strategies and problem-focused coping (Altınsoy and Aypay, 2023), as well as to enhanced feelings of self-compassion and shared humanity (Vazquez et al., 2021). These emotional skills are considered vital for an educators’ capacity to develop a prosocial classroom characterized by close, stimulating, supportive relationships with young children (Jennings and Greenberg, 2009). Previous studies have not considered post-traumatic growth and may provide an incomplete picture of the impact of ECEs’ adverse experiences because they fail to consider some of the strengths that individuals may develop in the aftermath of these experiences.

In summary, the current study was informed by a wealth of evidence that children’s early EF is fundamental to their healthy long-term development and resilience (Burchinal et al., 2020; de Maat et al., 2022). Theory and accumulating research also suggest that high quality early childhood education supports children’s EF development (Burchinal et al., 2008; Cadima et al., 2016) and that ECEs’ psychosocial stress and coping resources may affect their capacities to provide high-quality educational experiences (Jeon L. et al., 2019; Hubel et al., 2020). We aimed to explore the associations of ECEs’ experiences of early childhood adversity and post-traumatic growth with children’s EF, as reported independently by children’s parents. The novelty of this study lies in the simultaneous consideration of ECE’s ACEs and their post-traumatic growth. Assuming that educators’ experiences of post-traumatic growth may equip them with coping resources for managing their emotions and reduce their experience of psychological distress, in turn enabling them to better support children’s EF, we also examined whether associations of ECE ACEs and post-traumatic growth with children’s EF could be accounted by ECEs’ emotion regulation difficulties and psychological distress. Given the known link between measures of children’s socio-economic backgrounds and their EF (Lawson et al., 2018), we controlled for parent education in our analyses.

## 2 Method

### 2.1 Participants

Participants in the study were part of a larger sample of 163 early childhood professionals participating in an intervention study in Southeast Nebraska between 2019 and 2023 (University of Nebraska-Lincoln Human Subjects Committee approval number 20200720336EP; clinicaltrials.gov protocol ID 20336 “Testing the initial efficacy of a mindfulness compassion-based intervention to support well-being amongst early childhood educators”). We were able to recruit parents and children from classrooms of 53 of these ECEs, who formed the sample for reported analyses. This analytic sample of 53 ECEs were working in varying settings, including private and church-based preschools, and Educare and Head Start centers. All ECEs in the analytic sample who reported a gender were female and their mean age was 38 years (*SD* = 12.62, range = 21–70). Any facility providing early childcare for groups of children in Nebraska must be licensed and staff must complete at least 12 h of training in early learning standards and childcare safety each year. However, training varies, with many ECEs having undergraduate qualifications in early childhood development and related fields. In our sample, the mean length of education was 17.35 years (*SD* = 2.09, range = 14–21). The sample reflected a wide range of experience in early childcare, 31% of ECEs having been in the industry for <5 years, 24% for 6–10 years, 33% for 11–20 years, and 12% for >20 years. The majority (92%) of those who reported a race/ethnicity identified as White, 2% were Black/African American, 2% were Asian, 2% were Latinx/Hispanic, and 2% identified as more than one race. The median monthly household income bracket, reported on a 13-point scale, was $2,500 to $3,332. Note that the median monthly household income in Nebraska between 2018 and 2021 was $5,977 (US Census Bureau, 2022).

From the classrooms of these 53 ECEs, we recruited parents of 157 children aged 3–6 years (*M* = 4.38, *SD* = 0.75). The number of children recruited from each ECE’s classroom varied from 1 to 10. Fifty-three percent of these children were male and 47% were female. Seventy-six percent of the children were White, 4% were Black/African American, 3% were Asian, 12% were Latinx/Hispanic and 5% identified as another race. The breakdown of parent race/ethnicity was 76% White, 4% Black/African American, 3% Asian, 14% Latinx/Hispanic, and 3% Other race. The median (28% of sample) monthly household income bracket was $7,500 or more, although a range of incomes was reported and 32% of the sample reported household income levels < $2,500 per month.

### 2.2 Procedure

Teachers and parents provided written informed consent to participation in the study and all procedures were approved by a university human subjects protection committee. Teachers were recruited as part of a broader study to determine the effects of the Cultivating Healthy, Intentional, Mindful Educators (CHIME) intervention, a professional development curriculum focused on reflection, mindfulness and self-compassion in early childhood contexts (Hatton-Bowers et al., 2022). Prior to beginning CHIME, teachers completed a baseline survey via Qualtrics to assess their demographic characteristics, mindfulness, self-compassion, workplace and everyday stress, and psychological well-being. Teachers were sent an anonymous link via email so that they could complete the survey at a convenient time. Following teachers’ enrollment in the study, parents in their classrooms were recruited via emails and flyers. In the majority of cases (73%), parents were provided with a packet that included a paper consent form and paper-based surveys, which they either mailed back to investigators in a pre-paid envelope or brought back to the school for investigators to retrieve. The remaining parents completed the consent form via a Qualtrics link provided in the recruitment materials. After completing the consent form, these parents were automatically sent a link, via email, to the survey, which they completed online. All measures detailed below were included in the teacher or parent surveys.

### 2.3 Measures

#### 2.3.1 Early childhood educator measures

##### 2.3.1.1 Adverse Child Experiences

Teachers completed a 10-item version of the ACE survey (Felitti et al., 1998), which included statements such as, “Were your parents ever separated or divorced?” and “Did a household member go to prison?” Teachers responded yes (1) or no (0) to the statements and the total number of items was summed to create a total ACEs score. ACEs have been shown to assess a single construct and each cumulative increase in the ACE total score increases the odds of health and mental health morbidity (Dube et al., 2003; Bethell et al., 2017).

##### 2.3.1.2 Post-traumatic growth

Directly after completing the ACE survey, teachers were prompted to consider the most negative event that they had responded “yes” to on this survey. They were then asked to complete the 15-item Stress-Related Growth-Revised Scale in Boals and Schuler, (2018) relation to that adverse event. Items such as “As a result of this event, I experienced a change in the extent to which I can be myself and not try to be what others want me to be” are rated on a 7-item Likert scale from −3 (*a very negative change*) to 3 (*a very positive change*). If a teacher did not report any ACEs, they were allocated a 0 score for post-traumatic growth. A mean score of the items is used, with higher scores representing greater positive growth. This scale for measuring post-trauma growth shows strong convergent correlations with measures of wellbeing and mental health, as well as negative correlations with post-traumatic stress symptoms and avoidant coping (Boals and Schuler, 2018). Cronbach’s α internal reliability in the current sample was 0.95.

##### 2.3.1.3 Workplace stressors

The Child Care Work Job Stress Inventory—Job (Curbow et al., 2000) Demands subscale is a 17-item measure of job stress relating to parent interactions and challenging behaviors of children. Items include, “Parents come late to pick up their children” and “Children have behavior problems that are hard to deal with” on a 5-point Likert scale from 1 (*rarely/never*) to 5 (*most of the time*). While the measure typically has 17 items, the statement, “I feel pressure from my family to do a different kind of work” was omitted from the scale due to a technical error. Items are averaged together, with higher scores indicating greater job stress. This scale was carefully developed through focus groups with early childhood educators to identify their most common stressors in the workplace and scores corelate moderately with other widely used measures of workplace stress (Curbow et al., 2000). The subscale showed strong internal consistency in the current sample, Cronbach’s α = 0.84.

##### 2.3.1.4 Psychological distress

The Adult Outcome Questionnaire (Lambert et al., 1996) includes 45-items to measure DSM-based psychiatric symptoms and distress. Items such as, “I have trouble falling asleep or staying asleep” and “I feel worthless” are rated a 4-point Likert Scale from 0 (*never*) to 4 (*almost always*). The measure includes three subscales: symptom distress, social role, and interpersonal relations. Each subscale is summed, and the total score is created by summing each subscale with a range from 0 to 18. For ethical reasons, statements regarding suicide were removed from the survey. Due to this, the total score ranged from 0 to 176. The Outcome Questionnaire shows convergent correlations with measures of depression, anxiety and general psychological distress, as well as sensitivity to change in therapeutic contexts, supporting its validity (Lambert et al., 1996; Lambert, 2015). In this sample, Cronbach’s α = 0.83.

##### 2.3.1.5 Emotion regulation difficulties

The Difficulties in Emotion Regulation Scacle (DERS; Gratz and Roemer, 2004) is a 36-item measure that assesses emotion regulation difficulties. Items include, “I experience my emotions as overwhelming and out of control” and “I am confused about how I feel” rated on a 5-point Likert scale from 1 (*almost never*) to 5 (*almost always*). There are six subscales measuring: nonacceptance of emotional responses, difficulty engaging in goal-directed behavior, impulse control difficulties, lack of emotional awareness, limited access to emotion regulation strategies, and lack of emotional clarity. In this study, we used the composite, total score ranging from 36 to 180, with higher scores suggesting greater difficulties with emotion regulation. The DERS shows moderate correlations with similar measures and discriminates well between groups with low and high psychological distress, supporting its validity (Burton et al., 2022) In this sample, Cronbach’s α internal reliability = 0.88.

#### 2.3.2 Child measures

##### 2.3.2.1 Executive function

The Behavior Rating Inventory of Executive Function (BRIEF-P; Gioia et al, 2003) BRIEF-P is a parent-report measure of preschool-aged children’s EF. The 63-item measure includes statements such as, “Is impulsive” and “Resists change of routine, food, places, etc.,” and is rated on a 3-point scale of 1 (*never*), 2 (*sometimes*), or 3 (*often*). There are five subscales: inhibit, shift, emotional control, working memory, and plan and organize. Each subscale is summed to obtain a raw score and raw scores are converted to normative t-scores to create a Global Executive Composite score, as well as individual Inhibitory Control, Flexibility, and Emergent Metacognition scores. Importantly, higher scores on the BRIEF-P indicate greater child difficulties in this domain. BRIEF-P ratings show moderate correlations with neuropsychological assessments of EF and have been linked to children’s academic achievement, as well as to clinically significant symptoms of ADHD (Mahone and Hoffman, 2007; Clark et al., 2010; Soto et al., 2020). In this sample, Cronbach’s α = 0.95.

##### 2.3.2.2 Covariates

Demographic covariates were assessed via the teacher and parent surveys. Child age in years, child gender (1 = *male*), parent education in years, ECE years of teaching experience, and ECE monthly household income (on a scale of 1: ≤ $415 to 13: ≥ $7,500) were controlled for in all models. In addition, because it was possible that some parents completed the survey a short time after educators had begun CHIME intervention classes, we controlled for intervention group status (1 = CHIME Intervention, 0 = Wait-listed control group) in all models.

### 2.4 Analytic plan

Data were cleaned, scored, and analyzed in SPSS (Version 27.1; IBM Corp, 2020). After computing descriptive measures and Pearson’s correlations for primary analytic variables, we constructed mixed linear models to examine the relation of ECE ACES and post-traumatic growth to children’s BRIEF-P scores. These models included a random intercept for each ECE to account for the nesting of children within classrooms, used robust maximum likelihood estimation, and assumed a variance components covariance matrix structure for the random effects. Given the small sample size of teachers and the possibility of outlier influence when considering these self-reported experiences, we parametrically bootstrapped the mixed models to generate more robust 95% confidence intervals for the fixed effects. We performed 500 bootstrapped samples for each model, using a seed value to enable replication. Although a post-hoc power analysis indicated that we were underpowered to detect small effects (*R^2^* < 0.04) in a multi-level model, we elected to use this model because it accounts for the nesting of children within classrooms, which is important when examining the relation of teacher to child variables. However, repetition of our analyses using hierarchical linear regression yielded results similar to those reported below.

The first set of models examined the unique effect of teacher ACES on children’s self-regulation skills when accounting for demographic characteristics and teacher workplace stressors, as reported on the Child Care Work Job Stress Inventory. The second set of models examined the unique effect of ECEs’ post-traumatic growth on children’s EF after accounting for any ECE ACEs and demographic characteristics. The third set of models examined the relation of teacher post-traumatic growth to children’s EF only among the teachers who reported at least one ACE (*n* = 28). In the final model, we again examined teacher ACE scores and post-traumatic growth in relation to children’s EF after accounting for teacher emotion regulation difficulties, measured on the DERS, and teacher psychological distress, measured using the Adult Outcome Questionnaire. Some teachers were missing covariate data and therefore were not able to be included in the analytic models. Specifically, 5 teachers did not report a household income, 2 did not report years of experience and 2 did not report job stress, leaving a total of 48 teachers and 138 children for the models. Re-running analyses with multiply imputed data for these covariates produced similar results to those reported in the manuscript. A *p*-value of 0.05 was used to assess statistical significance and bootstrapped confidence intervals that did not include 0 also are highlighted.

## 3 Results

Within this sample of ECEs, 28 (53%) reported experiencing at least one ACE. Of those who did report ACEs, 11 (21%) reported one ACE, whereas 10 (19%) reported 3 or more ACEs. The most commonly reported ACE was household problem drinking/alcoholism (22%), whereas the least common were household member incarceration (2%) and food insecurity/inability to afford care (2%, see Supplementary materials). The average level of post-traumatic growth among those reporting ACEs was 0.88 (*SD* = 0.93). On this scale, the endorsement of 0 indicates no traumatic growth, whereas the endorsement of a rating of one indicates some traumatic growth, indicating that these teachers, on average, felt that they had experienced some growth after these adverse events.

Table 1 describes the descriptive statistics and bivariate associations between educator ACEs, post-traumatic growth, workplace stressors, emotion regulation and psychological distress, and child measures of EF. Educators with a higher number of ACEs reported increased difficulties with emotion regulation, as well as increased psychological distress. The only significant bivariate association between these ECE characteristics and child EF was for educator ACEs, where a higher number of ACEs related to increased child difficulties with flexibility, as reported by parents. Note that a score of 63 or more on the Adult Outcome Questionnaire is considered clinically significant (Lambert, 2015). Although the average ECE in our sample did not meet this cut-off, 27% of the sample did reach this cut-off criterion. Within the group of educators who reported an ACE, the correlation of educator post-trauma growth with child General EF Difficulties was *r* = −0.24, *p* = 0.04. Correlations were of similar magnitude for the Inhibitory Self-Control (*r* = −0.28, *p* = 0.02), Flexibility (*r* = −0.22, *p* = 0.06) and Emergent Metacognition (*r* = −0.18, *p* = 0.11) scales.

**Table 1:** Descriptive statistics and correlations between teacher and child variables.

	1	2	3	4	5	6	7	8	9	10	11	12	13	14	15	16
*M*	1.28	0.53	0.46	0.66	4.38	0.53	17.08	1.56	8.00	48.47	49.16	74.11	5.96	5.82	49.71	51.52
*SD*	1.71	0.50	0.80	0.48	0.75	0.50	3.74	6.34	2.78	11.11	21.86	2.18	1.71	1.08	1.07	11.20
1. Total ACES																
2. ACE or not	0.71^**^															
3. Post-traumatic growth	0.45^**^	0.56^**^														
4. Intervention group	0.12	0.08	0.05													
5. Child age	0.05	0.04	0.09	−0.16												
6. Child male gender	0.06	0.05	0.01	0.06	−0.07											
7. Parent education	−0.01	−0.08	−0.04	−0.14	0.10	−0.07										
8. ECE Yrs experience	−0.17	−0.17	0.08	−0.35^*^	0.24	−0.23	0.26									
9. ECE income	−0.18	−0.12	−0.04	−0.07	0.59^**^	−0.18	0.08	0.38^**^								
1. ECE workplace stressors	0.21	0.12	0.03	0.33^*^	−0.13	0.25	−0.10	−0.11	−0.29^*^							
11. ECE Psychological distress	0.31^*^	0.06	−0.27	0.14	−0.17	0.05	−0.07	−0.06	−0.16	0.54^**^						
12. ECE DERS	0.35^*^	0.15	−0.21	0.22	−0.08	0.25	−0.27	−0.31^*^	−0.23	0.47^**^	0.72^**^					
13. BRIEF-P general EF	0.16	0.07	−0.10	0.12	−0.03	0.25^**^	−0.11	−0.12	−0.11	0.00	0.04	0.01				
14. BRIEF-P inhibitory self control	0.13	0.06	−0.13	0.11	−0.04	0.24^**^	−0.06	−0.11	−0.13	0.06	0.13	0.07	0.91^**^			
15. BRIEF-P flexibility	0.14	0.15	−0.03	0.07	0.02	0.27^**^	0.01	−0.13	−0.04	−0.09	0.01	−0.03	0.83^**^	0.80^**^		
16. BRIEF-P emergent metacognition	0.07	0.01	−0.11	0.17*	−0.03	0.24^**^	−0.15	−0.07	−0.10	0.01	0.01	−0.01	0.91^**^	0.72^**^	0.62^**^	-

Correlations between ECE variables are within the ECE sample (*n* = 53). Correlations with BRIEF-P variables include the full child sample (*n* = 157). Early childhood educators who did not report an ACE were allocated a post-traumatic growth score of 0.

Yrs, Years; ECE, Early childhood educator; DERS, Difficulties in Emotion Regulation Scale; Workplace stressors measured using Child Care Worker Job Stress Inventory; psychological distress measured using the Adult Outcome Questionnaire; **p < 0.01, *p < 0.05.

The intra-class correlations for unconditional mixed effects models indicated that the nested effects of ECE grouping on children’s BRIEF-P scores were small. Specifically, nesting effects accounted for 8% of the variance in children’s Composite EF, 11% of the variance in Inhibitory Self Control, 6% of the variance in Flexibility, and 8% of the variance in children’s Emergent Metacognition BRIEF-P scores.

Table 2 describes the first set of models examining relations of ECE ACEs to children’s EF after accounting for several teacher and family background characteristics, as well as teacher workplace stressors. ACEs were not associated with any of the child EF scale scores after accounting for multiple background characteristics, with neither raw nor bootstrapped model estimates emerging as significant. Parents reported higher EF difficulties for males across all BRIEF-P scales (*p*’s < 0.05). A similar pattern of findings held when considering whether teachers had experienced any ACEs (i.e., treating ACEs as a binary yes/no variable rather than a total score, see Supplementary materials).

**Table 2:** Relations of ECE adverse childhood experiences to children’s executive function.

	BRIEF-P general EF				BRIEF-P inhibitory self control				BRIEF-P flexibility				BRIEF-P emergent metacognition			
		Est	SE	Bootstrapped CI	Est	SE	Bootstrapped CI		Est	SE	Bootstrapped CI		Est	SE	Bootstrapped CI	
*Fixed effects*																
Intercept	58.91	9.41	41.96	73.75	56.41	7.61	4.01	7.13	57.83	7.70	4.81	71.58	57.79	8.81	39.37	74.15
Intervention group	2.49	2.55	−1.69	6.42	2.37	2.06	−1.98	6.78	1.45	1.81	−2.10	4.79	4.20	2.18	0.02	8.42
Child age	0.04	1.28	−2.03	3.24	−0.01	1.21	−1.45	3.40	−0.06	1.22	−1.85	2.98	0.10	1.45	−2.63	3.02
Child male gender	5.23**	1.76	**1.96**	**9.07**	4.44*	1.73	**0.80**	7.85	4.78*	1.63	1.24	7.79	5.78**	1.84	2.69	9.90
Parent education	−0.10	0.24	−0.75	0.38	−0.02	0.24	−0.56	0.39	0.22	0.20	−0.24	0.58	−0.21	0.31	−0.89	0.37
ECE Yrs experience	−0.08	0.19	−0.40	0.23	−0.03	0.16	−0.35	0.29	−0.19	0.13	−0.44	0.07	0.05	0.16	−0.27	0.40
ECE income	−0.53	0.42	−1.25	0.02	−0.62	0.33	−1.36	−0.10	−0.30	0.32	−1.05	0.23	−0.53	0.37	−1.29	0.14
ECE workplace stressors	−0.13	0.11	−0.28	0.04	−0.08	0.08	−0.22	0.09	−0.23	0.08	−0.39	−0.07	−0.11	0.09	−0.29	0.07
ECE total ACEs	0.17	0.73	−1.02	1.70	0.08	0.61	−1.05	1.44	0.13	0.57	−0.74	1.45	−0.09	0.73	−1.34	1.50
*Random effects*																
Residual	94.89	13.30			82.44	11.66			80.29	9.93			105.19	14.54		
Intercept (ECE)	14.33	10.26			17.48	12.68			12.33	12.47			15.38	14.98		
−2 Restricted log likelihood	1020.33				1006.29				998.97				1033.28			
AIC	1024.33				1010.29				1002.97				1037.28			
Pseudo-*R*^2^	0.09				0.09				0.10				0.10			

Bolded confidence intervals represent significant effects in bootstrapped analyses; **p < 0.01, *p < 0.05 in non-bootstrapped models. *N* = 48 teachers, 138 children. BRIEF-P, Behavior Rating Inventory of Executive Function-Preschool; Yrs, Years; ECE, Early childhood educator; AIC, Akaike’s Information Criterion; Est, Estimate. Pseudo R^2^ is marginal and reflects the variance attributable to the fixed effects.

Table 3 shows estimates from the second set of models examining the relation of ECEs’ post-traumatic growth to child EF after accounting for ECE ACE scores. These analyses showed a negative association between ECE post-traumatic growth and children’s EF difficulties. Specifically, higher post-traumatic growth was linked to fewer child difficulties with general EF (*p* = 0.02), inhibitory control (*p* = 0.03), and metacognition (*p* = 0.03). Counter to expectations, educators’ perceived workplace stressors were linked to fewer child flexibility difficulties after accounting for demographic factors and ACEs (*p* = 0.02). Collectively, the fixed effects predictors in these models explained 11–14% of the variance in children’s BRIEF-P scores.

**Table 3:** Relations of early educators’ post-trauma growth to children’s executive function.

	BRIEF-P general EF				BRIEF-P inhibitory self control				BRIEF-P flexibility				BRIEF-P emergent metacognition			
	Est	SE	Bootstrapped CI		Est	SE	Bootstrapped CI		Est	SE	Bootstrapped CI		Est	SE	Bootstrapped CI	
*Fixed effects*																
Intercept	63.18	8.38	44.42	76.45	59.75	7.61	42.49	72.63	59.71	8.61	42.44	76.97	62.21	9.02	41.34	77.69
Intervention group	3.32	2.08	−0.77	7.31	3.02	2.08	−0.98	7.58	1.90	2.34	−2.84	6.63	5.08	2.15	0.82	9.26
Child age	−0.11	1.30	−2.04	3.18	−0.18	1.19	−1.44	3.23	−0.12	1.19	−2.47	2.23	0.08	1.43	−2.62	2.99
Child male gender	5.00**	1.74	**1.79**	**8.87**	4.26*	1.70	**0.82**	**7.72**	4.71**	1.64	**1.49**	**7.92**	5.51**	1.82	**2.57**	**9.78**
Parent education	−0.13	0.27	−0.72	0.36	−0.03	0.24	−0.54	0.37	0.22	0.22	−0.22	0.66	−0.25	0.30	−0.89	0.34
ECE yrs. experience	**0.04**	0.16	−0.28	0.36	0.08	0.16	−0.22	0.39	−0.12	0.18	−0.48	0.25	0.17	0.17	−0.17	0.52
ECE income	−0.59	0.34	−1.33	**−0.03**	−0.65	0.33	**−1.43**	**−0.15**	−0.34	0.38	−1.13	0.45	−0.62	0.36	−1.35	0.02
ECE workplace stressors	−0.20	0.08	−0.33	−0.04	−0.14	0.08	−0.28	0.02	−0.26*	0.11	**−0.48**	**−0.05**	−0.20	0.09	−0.36	0.01
ECE ACE score	1.27	0.81	−0.30	2.97	1.13	0.69	−0.32	2.57	0.70	0.81	−0.92	2.32	1.00	0.87	−0.66	2.86
ECE post-traumatic growth	−4.22*	1.30	**−6.50**	**−1.23**	−4.0*	1.09	**−5.94**	**−1.69**	−2.17	1.73	−5.70	1.35	−4.26*	1.90	**−8.28**	**−1.01**
*Random effects*																
Residual	96.04	12.35			83.67	12.50			81.35	9.99			106.90	14.97		
Intercept (ECE)	7.76	13.55			10.97	11.27			1.07	12.51			8.18	9.86		
−2 Restricted log likelihood	1012.43				998.65				994.52				1025.65			
AIC	1016.53				1002.65				998.52				1029.65			
Pseudo-R^2^	0.13				0.12				0.11				0.14			

Bolded confidence intervals represent significant effects in bootstrapped analyses; **p < 0.01, *p < 0.05 in non-bootstrapped models. *N* = 48 teachers, 138 children. BRIEF-P, Behavior Rating Inventory of Executive Function-Preschool; Yrs, Years; ECE, Early childhood educator; AIC, Akaike’s Information Criterion; Est, Estimate. Pseudo R^2^ is marginal and reflects the variance attributable to the fixed effects.

Given that post-traumatic growth was, by definition, limited to educators who reported an ACE, the third set of models was conducted only within this smaller subset of teachers (*n* = 28). Table 4 shows that, within this group of educators, post-traumatic growth related to fewer child general EF difficulties (*p* = 0.02) and fewer difficulties with metacognition (*p* = 0.02). Although non-bootstrapped estimates were not significant for the Inhibitory Self Control (*p* = 0.07) or Flexibility (*p* = 0.05) scales, bootstrapped confidence intervals indicated significant associations of post-traumatic growth with all BRIEF-P scales within this subsample of ECEs. These fixed effects accounted for 16–21% of the variance in children’s EF.

**Table 4:** Relations of educators’ post-traumatic growth to children’s EF within the group of early childhood educators who reported at least one adverse childhood experience.

	BRIEF-P general EF				BRIEF-P inhibitory self control				BRIEF-P flexibility				BRIEF-P emergent metacognition			
	Est	SE	Bootstrapped CI		Est	SE	Bootstrapped CI		Est	SE	Bootstrapped CI		Est	SE	Bootstrapped CI	
*Fixed effects*																
Intercept	67.74	11.90	4.29	87.75	65.57	9.99	43.10	81.84	67.55	11.03	4.07	85.27	64.47	12.62	37.28	86.25
Intervention group	4.82	2.87	−1.34	1.37	3.58	2.81	−2.30	9.06	4.08	2.76	−1.09	9.82	7.14	3	0.64	13.57
Child age	−1.51	2.87	−5.34	5.94	−0.30	2.37	−3.46	6.27	−1.32	2.32	−4.65	4.62	−1.86	2.94	−6.25	5.51
Child male gender	3.51	2.78	−1.90	8.77	3.79	2.70	−1.71	8.99	3.04	2.95	−3.43	8.78	4.57	2.78	−0.56	9.85
Parent education	0.38	0.37	−0.47	1.04	0.40	0.34	−0.25	1.09	0.37	0.33	−0.31	0.94	0.29	0.41	−0.79	0.95
ECE Yrs experience	0.27	0.26	−0.21	0.84	0.18	0.24	−0.28	0.67	0.18	0.24	−0.26	0.65	0.47	0.28	−0.04	1.09
ECE income	−0.74	0.80	−2.68	0.40	−1.31	0.73	**−3.10**	**−0.35**	−0.52	0.66	−2.15	0.47	−0.44	0.85	−2.36	0.99
ECE workplace stressors	−0.28	0.12	**−0.52**	**−0.08**	−0.24	0.11	**−0.48**	**−0.05**	−0.32	0.13	**−0.56**	**−0.05**	−0.30	0.13	**−0.58**	**−0.05**
ECE post-traumatic growth	−5.73*	1.90	**−9.53**	**−2.08**	−4.78	1.62	**−8.17**	**−1.98**	−4.82	1.82	**−8.79**	**−1.64**	−5.73*	2.24	**−1.26**	**−1.43**
*Random effects*																
Residual	105.55	21.81			87.30	2.62			89.67	17.31			115.13	24.40		
Intercept (ECE)	9.04	29.82			20.31	28.13			11.509	25.83			7.85	29.40		
−2 Restricted Log Likelihood	491.04				483.96				482.73				495.60			
AIC	495.26				487.96				486.73				499.60			
Pseudo-*R*^2^	0.18				0.18				0.16				0.21			

Bolded confidence intervals represent significant effects in bootstrapped analyses; **p < 0.01, *p < 0.05 in non-bootstrapped models. *N* = 24 teachers, 68 children. BRIEF-P, Behavior Rating Inventory of Executive Function-Preschool; Yrs, Years; ECE, Early childhood educator; AIC, Akaike’s Information Criterion; Est, Estimate.

In the final set of models, we examined whether the effects of post-traumatic growth were retained after accounting for teachers’ emotion regulation difficulties and psychological distress. Specifically, we entered DERS and Adult Outcome Questionnaire scores into models for the full sample of ECEs. As shown in Table 5, after accounting for these educator psychological variables, the association of educator post-traumatic growth with children’s general EF was attenuated (*p* = 0.07), although bootstrapped confidence intervals continued to support a significant association. Similarly, the correlation of post-traumatic growth with the Inhibitory Self Control (*p* = 0.18) scale was attenuated, although bootstrapped confidence intervals still indicated significant associations. The association of post-traumatic growth with metacognition remained significant, where higher post-traumatic growth correlated with fewer parent-reported difficulties (*p* = 0.05). Bootstrapped confidence intervals also supported a positive association of educator income with child EF. ECE emotion dysregulation difficulties and psychological distress were not related to children’s EF. When post-traumatic growth was removed from the same models, ACEs again showed no association with BRIEF-P scores and psychological distress was only significant in relation to children’s Inhibitory Self Control scores (*p* = 0.03), with no other significant links emerging for these ECE psychological variables (see Supplementary materials).

**Table 5:** Relations of early educators’ adverse experiences and post-traumatic growth to children’s EF after accounting for ECE emotion regulation and psychological distress.

	BRIEF-P general EF				BRIEF-P inhibitory self control				BRIEF-P flexibility				BRIEF-P emergent metacognition			
	Est	SE	Bootstrapped CI		Est	SE	Bootstrapped CI		Est	SE	Bootstrapped CI		Est	SE	Bootstrapped CI	
*Fixed effects*																
Intercept	64.96	8.92	45.34	79.46	61.04	8.07	43.45	74.60	61.87	8.36	44.55	77.17	63.82	9.56	41.90	8.22
Intervention group	3.52	2.04	−0.56	7.57	3.29	2.03	−0.61	7.56	2.09	1.87	−1.62	5.53	5.24*	2.15	**0.94**	**9.38**
Child age	0.39	1.40	−1.74	3.73	0.67	1.25	−1.07	4.00	0.45	1.28	−1.63	3.61	0.27	1.55	−2.89	3.46
Child male gender	5.07**	1.76	**1.89**	**8.94**	4.35**	1.70	**0.84**	**7.85**	4.77**	1.64	**1.41**	**7.79**	5.58**	1.85	**2.51**	**9.86**
Parent education	−0.16	0.27	−0.74	0.35	−0.08	0.24	−0.58	0.36	0.18	0.20	−0.27	0.55	−0.26	0.30	−0.90	0.34
ECE Yrs. experience	−0.09	0.18	−0.44	0.26	−0.05	0.17	−0.40	0.28	−0.27	0.15	−0.56	0.02	0.07	0.19	−0.30	0.46
ECE income	−0.59	0.34	**−1.36**	**−0.03**	−0.74	0.33	**−1.49**	**−0.21**	−0.35	0.32	−1.10	0.19	−0.58	0.37	−1.34	0.08
ECE workplace stressors	−0.19	0.09	**−0.39**	**−0.03**	−0.21	0.09	**−0.40**	**−0.05**	−0.27	0.10	**−0.47**	**−0.10**	−0.15	0.11	−0.38	0.07
ECE DERS	−0.09	0.06	−0.20	0.03	−0.08	0.05	−0.18	0.04	−0.10	0.06	−0.21	0.02	−0.08	0.07	−0.21	0.06
ECE psychological distress	0.08	0.06	−0.02	0.23	0.13	0.06	**0.06**	**0.27**	0.10	0.06	−0.01	0.24	0.03	0.07	−0.10	0.19
ECE total ACEs	1.15	0.84	−0.44	2.95	0.64	0.69	−0.82	2.05	0.50	0.75	−0.84	2.05	1.09	0.90	−0.55	3.16
ECE post-traumatic growth	−3.63	1.53	**−6.44**	**−0.20**	−2.72	1.30	−4.82	0.29	−1.43	1.39	−4.10	1.52	−4.14	1.71	**−7.04**	**−0.25**
*Random effects*																
Residual	95.63	13.59			80.78	11.95			85.25	13.76			105.47	16.33		
Intercept (ECE)	8.96	1.21			15.02	11.93			9.94	12.96			9.73	12.96		
−2 Restricted log likelihood	1017.87				1002.79				999.49				1031.48			
AIC	1021.87				1006.79				1003.49				1035.48			
Pseudo-R^2^	0.14				0.14				0.13				0.17			

Bolded confidence intervals represent significant effects in bootstrapped analyses; **p < 0.01, *p < 0.05 in non-bootstrapped models. *N* = 48 teachers, 138 children. BRIEF-P, Behavior Rating Inventory of Executive Function-Preschool; Yrs, Years; ECE, Early childhood educator; DERS, Difficulties in Emotion Regulation Scale; AIC, Akaike’s Information Criterion; Est, Estimate.

## 4 Discussion

Accumulating research highlights the impact of ECEs’ psychosocial stressors and resources on their classroom practice and on children’s development of fundamental socio-emotional competencies (Buettner et al., 2016; Jeon L. et al., 2019). Executive function develops rapidly during early childhood and acts as a lifelong resource for resilience, supporting children’s capacity to manage their behavior and emotions, including in the context of stress (de Maat et al., 2022; Zhou et al., 2022). In this study, we examined the relations of ECEs’ experiences of adverse events in their own childhoods, and their post-traumatic growth after these experiences, to the EF of children in their classrooms. Educators’ reports of post-traumatic growth after adverse childhood events generally correlated with more favorable parent ratings of children’s EF after accounting for several background variables and current educator workplace stressors. In contrast, ECEs’ experiences of ACEs were not associated with children’s EF after accounting for demographic variables. These findings suggest that early childhood educators’ experiences of post-trauma growth may act as resources, helping them to foster children’s EF and associated life-long resilience.

Our study showed no significant associations between educators’ experiences of ACEs and children’s EF. While higher ACE scores did correlate with higher levels of psychological distress and higher emotion dysregulation among ECEs, these latter variables also generally were not associated with children’s EF. It is possible that other, unmeasured factors, such as higher levels of community trauma and stress or the collective stress of all educators in the classroom, may play a role. Further research considering a broader set of measures coupled with observations of ECEs’ interactions with children will be necessary to better understand the implications of ECE ACEs for children’s outcomes.

In this study, ECE reports of ACEs were lower than in previous studies both of ECEs and of community samples (Hubel et al., 2020; Ports et al., 2020). There was an especially low prevalence of household financial insecurity or imprisonment. These low rates are somewhat surprising, given that the sample represented a range of income levels and early education contexts. It is possible that these low rates reflect selection bias, where those educators with relatively lower adverse experiences were more able to participate in this study and had parents in their classrooms that participated in the study. Regardless, there was a wide range of reported experiences, with 19% of the sample reporting 3 or more ACEs.

Importantly, after controlling for a number of background characteristics, ECEs’ reports of post-traumatic growth related to fewer parent-reported child difficulties with general EF, inhibitory self-control and metacognition. This novel finding suggests that those educators who experience changes in their sense of self, their philosophy on life, and their relationships with others as a result of traumatic and negative childhood events may have developed resources that equip them to engage with children in ways that support children’s EF. However, specific educator resources were not studied here and there may be multiple mechanisms or pathways via which the link between teacher post-trauma growth and child EF emerges. It is possible, for instance, that those who report post-traumatic growth also have personality characteristics, e.g., an optimistic outlook, or increased social support networks, linked both to the likelihood of experiencing post-traumatic growth and to the EF of children in their classrooms (Casali et al., 2022). It is also possible that educators who have experienced these especially challenging childhood events have developed deeper empathy, connection, and compassion for others as a result of their experiences (Lim and DeSteno, 2016), enabling them to support children’s EF even when they personally may experience psychological distress. Our findings diverge from those of a recent study showing that ECEs’ ACES correlated with more negative teacher-child interactions and with higher rates of child challenging behavior in the classroom, whereas ECEs’ self-reported resilience did not (Rancher and Moreland, 2023). It is possible that this reflects differences in the populations studied, with 70% of Racher and Moreland’s study’s sample having experienced ACEs. The resilience scale used in their study also assessed aspects of emotion regulation that, in our study, were not associated with child EF. Finally, we examined post-traumatic growth specifically in relation to educator ACEs, whereas Rancher and Moreland focused on resilience across their full sample, independent of educator experiences of adversity. By definition, post-traumatic growth is a response to trauma (Tedeschi and Calhoun, 1996) and it may be especially important for those who have experienced adverse, challenging events.

It is also important to note other associations and null findings in this study. First, after accounting for multiple other variables, bootstrapped models suggested that ECEs’ monthly household income was associated with all EF scales, where higher educator income related to higher child EF. These findings echo previous studies showing that educator wages are associated with observed quality of instruction (Johnson et al., 2021) and underscore the importance of fair compensation for caregivers charged with supporting children’s development during this critical early childhood period. Unexpectedly, educators’ emotion regulation difficulties and psychological distress were not significantly associated with children’s EF. Workplace stressors, when they did show associations, actually related to fewer parent-reported child EF difficulties. These null findings do not support the prosocial classroom model, perhaps suggesting that other factors play a more crucial role in helping educators to create classroom environments supportive of children’s EF development. While surprising, these findings are not unprecedented; previous studies have reported similarly weak associations between ECEs’ levels of depression and workplace stressors and their teaching practices (Roberts et al., 2016; Rancher and Moreland, 2023).

Limitations of the study include the small sample size; use of parent report, as opposed to direct, assessments of children’s EF; cross-sectional design; lack of direct assessments of classroom quality; and lack of equivalent measures of parent ACEs and post-trauma growth. In addition, despite the strong utility of ACEs for predicting adults’ health and psychological well-being, these items do not represent the full gamut of negative or traumatic experiences occurring in people’s lives, neglecting factors such as racial trauma or exposure to terrorism (McEwen and Gregerson, 2019). The assumption of the ACE scale is that effects of these stressors are cumulative, with each type of trauma assumed to have equal weighting. However, there have been calls for more finessed approaches that recognize the potential variability in the impact of these different types of trauma (Lacey and Minnis, 2020). Moreover, ACE measurement in adulthood relies on retrospective recall, which may be difficult for events experienced early in life and which may also be affected by subsequent interpretation of those events. It is possible, too, that ECEs may have experienced more recent traumatic events as adults, which could amplify or confound the impact of ACEs.

It is important to emphasize that the phenomenon of post-traumatic growth is complex and multi-determined and may involve personality characteristics present before the adverse event occurred, as well as the individual’s access to social and instrumental supports that allow them the time and space for assimilation and accommodation of these especially difficult experiences (Joseph et al., 2012; Jayawickreme and Blackie, 2014). As illustrated in this study, post-traumatic growth was coincident with the number of adverse experiences educators reported and adverse experiences were linked to higher levels of psychological distress and emotional dysregulation among educators. Individuals who report growth experiences after adverse events may simultaneously experience high levels of distress while still recognizing some positive implications of their traumatic experiences. It may therefore be reductionistic to assume that a single strategy of supporting growth may alleviate the impact of these adverse experiences on educator well-being. Equally, the study findings argue against a ‘deficit model’ that assumes that ECEs who have experienced adverse childhood events are less effective at supporting children’s EF. Indeed, the relations of ACEs to children’s EF were minimal and generally were not significant after accounting for several other stressors. This suggests that, while post-traumatic growth may boost educators’ resources for supporting children’s EF, the experience of distress and emotional dysregulation may not necessarily impinge on the quality of care they are able to provide.

Findings suggest that psychological supports for ECEs to process and reflect on their traumatic experiences may be of value in early childhood settings, especially for those ECEs supporting children from marginalized communities at disproportionate likelihood of experiencing their own ACEs (Ports et al., 2020). Supports may take the form of access to mental health practitioners who can support ECEs to process trauma, as well as increased financial compensation to alleviate financial insecurity associated with an undervalued and low-paying profession (Whitebrook et al., 2016). Increased access to professional development opportunities that encourage the development of buffering psychological resources may also be beneficial. For instance, in their study of educators in Head Start, Whitaker et al. (2014) reported that educators’ dispositional mindfulness buffered the impact of ACEs on their health. Interventions focused on mindfulness may therefore be beneficial. An encouraging recent study reported that a well-being intervention involving coaching, relaxation, nutrition, and physical activity was especially beneficial in improving the classroom-based positive affect of ECEs with high ACE levels (Stone et al., 2023). In a systematic review of trauma-informed interventions delivered to ECEs, there were positive benefits to ECE’s trauma awareness, knowledge and practices (Sun et al., 2023). However, many interventions focus on understanding young children’s experience of trauma. It may be beneficial to include supports for ECEs to process and learn strategies for healing from their own ACEs as part of these interventions.

Although exploratory and in need of careful replication in larger, more diverse samples, novel findings from this study suggest that ECEs’ early life experiences and psychological resources arising from those experiences may have inter-generational implications for human well-being. Supporting ECEs to process their own childhood trauma in desired places that are perceived to be safe and helpful (e.g., therapists, groups, culturally relevant practices) could potentially offer a promising means to enhance their own mental health, as well as children’s EF and associated resilience through the lifespan.

## Data availability statement

The raw data supporting the conclusions of this article will be made available by the authors, without undue reservation.

## Ethics statement

The studies involving humans were approved by University of Nebraska Institutional Review Board Human Research Subjects Protection Program. The studies were conducted in accordance with the local legislation and institutional requirements. Written informed consent for participation in this study was provided by the participants’ legal guardians/next of kin.

## Author contributions

CC: Conceptualization, Formal analysis, Funding acquisition, Methodology, Project administration, Supervision, Writing – original draft. HH-B: Conceptualization, Funding acquisition, Project administration, Supervision, Writing – review & editing. KA: Data curation, Writing – original draft. SR: Data curation, Writing – review & editing. GP: Conceptualization, Funding acquisition, Supervision, Writing – review & editing.
