# Supplementary material for: Growing through adversity: the relation of early childhood educator post-traumatic growth to young children’s executive function
Source: Front Psychol. 2024 Mar 13;15:1272294. doi: 10.3389/fpsyg.2024.1272294 (PMC10965675; doi:10.3389/fpsyg.2024.1272294)
Supplement: Supplementary file 1 [file Data_Sheet_1.docx]

Supplementary Material: Growing through adversity: The relation of early childhood educator post-traumatic growth to young children’s executive function

# Supplementary Tables

Supplementary Table 1: Proportion of early childhood educators (n = 53) reporting different adverse childhood experiences

|  | % Reported ACE |
| --- | --- |
| Adult swore, insulted, humiliated | 16 |
| Adult pushed, slapped, threw, hit | 8 |
| Adult touched in sexual way/attempted intercourse | 14 |
| Felt unloved/uncared for | 16 |
| Not enough to eat/not protected | 2 |
| Parents separated/divorced | 20 |
| Mother pushed, slapped, hit | 8 |
| Household problem drinker/alcoholic | 22 |
| Household depression, mental illness, suicide | 10 |
| Household member incarcerated | 2 |

Supplementary Table 2: Relation of ECE experience of any ACE to children’s executive function

|  | General EF Composite | | | | Inhibitory Self Control | | | | Flexibility | | | | Emergent Metacognition | | | |
| --- | --- | --- | --- | --- | --- | --- | --- | --- | --- | --- | --- | --- | --- | --- | --- | --- |
|  | Est | SE | Bootstrapped CI | | Est | SE | Bootstrapped CI | | Est | SE | Bootstrapped CI | | Est | SE | Bootstrapped CI | |
| *Fixed effects* |  |  |  |  |  |  |  |  |  |  |  |  |  |  |  |  |
| Intercept | 58.94 | 8.13 | 41.74 | 73.41 | 56.45 | 7.69 | 39.59 | 70.58 | 56.37 | 7.87 | 39.29 | 70.17 | 58.82 | 8.85 | 39.90 | 75.59 |
| Intervention group | 2.46 | 2.04 | -1.72 | 6.32 | 2.36 | 2.03 | -1.78 | 6.69 | 1.46 | 1.77 | -2.21 | 4.75 | 4.21 | 2.13 | **.25** | **8.31** |
| Child age | .04 | 1.32 | -2.02 | 3.23 | -.01 | 1.21 | -1.43 | 3.41 | -.12 | 1.22 | -1.88 | 2.98 | .12 | 1.44 | -2.64 | 3.04 |
| Child male gender | 5.22** | 1.78 | **1.88** | **9.02** | 4.44* | 1.73 | **.78** | **7.85** | 4.70** | 1.62 | **1.22** | **7.72** | 5.82** | 1.85 | **2.69** | **9.92** |
| Parent education | -.09 | .27 | -.73 | .38 | -.02 | .24 | -.56 | .40 | .23 | .20 | -.24 | .57 | -.22 | .30 | -.88 | .37 |
| ECE Yrs experience | -.08 | .16 | -.41 | .24 | -.03 | .16 | -.36 | .29 | -.18 | .14 | -.43 | .09 | .04 | .17 | -.28 | .40 |
| ECE Income | -.54 | .34 | -1.25 | .02 | -.63 | .33 | -1.38 | -.10 | -.28 | .32 | -1.02 | .28 | -.54 | .37 | -1.31 | .13 |
| ECE Workplace stressors | -.12 | .07 | -.28 | .03 | -.07 | .08 | -.21 | .09 | -.22* | .08 | **-.38** | **-.05** | -.12 | .09 | -.30 | .06 |
| ECE Experienced ACE (1 = Yes, 0 = No) | .21 | 1.54 | -2.59 | 3.56 | .07 | 1.49 | -2.70 | 3.40 | 1.99 | 1.48 | -.62 | 5.19 | -1.26 | 1.72 | -4.20 | 2.33 |
| *Random effects* |  |  |  |  |  |  |  |  |  |  |  |  |  |  |  |  |
| Residual | 94.93 | 12.344 |  |  | 82.40 | 11.65 |  |  | 79.95 | 9.91 |  |  | 105.28 | 14.56 |  |  |
| ECE | 14.31 | 13.97 |  |  | 17.57 | 12.69 |  |  | 11.76 | 12.28 |  |  | 14.79 | 15.02 |  |  |
|  |  |  |  |  |  |  |  |  |  |  |  |  |  |  |  |  |
| -2 Restricted Log Likelihood | 1018.24 |  |  |  | 1004.16 |  |  |  | 999.82 |  |  |  | 1034.84 |  |  |  |
| AIC | 1022.24 |  |  |  | 1008.16 |  |  |  | 999.92 |  |  |  | 1034.94 |  |  |  |
|  |  |  |  |  |  |  |  |  |  |  |  |  |  |  |  |  |
| Pseudo-R^2^ | .09 |  |  |  | .07 |  |  |  | .11 |  |  |  | .10 |  |  |  |

Note: Bolded confidence intervals represent significant effects in bootstrapped analyses; ** *p* < .01, **p* < .05 in non-bootstrapped models. Teacher n = 51, child n = BRIEF-P: Behavior Rating Inventory of Executive Function- Preschool, Yrs: Years, ECE: Early childhood educator, AIC: Akaike’s Information Criterion.

Supplementary Table 3: Relation of educators’ adverse experiences to children’s executive function after accounting for emotion regulation and psychological distress

|  | BRIEF-P General EF Composite | | | | BRIEF-P Inhibitory Self Control | | | | BRIEF-P Flexibility | | | | BRIEF-P Emergent Metacognition | | | |
| --- | --- | --- | --- | --- | --- | --- | --- | --- | --- | --- | --- | --- | --- | --- | --- | --- |
|  | Est | SE | Bootstrapped CI | | Est | SE | Bootstrapped CI | | Est | SE | Bootstrapped CI | | Est | SE | Bootstrapped CI | |
| *Fixed effects* |  |  |  |  |  |  |  |  |  |  |  |  |  |  |  |  |
| Intercept | 62.07 | 8.55 | 44.47 | 77.23 | 59.32 | 7.81 | 42.56 | 73.14 | 60.97 | 8.05 | 44.28 | 76.07 | 60.45 | 9.17 | 40.69 | 78.00 |
| Intervention group | 2.94 | 2.04 | -1.20 | 6.67 | 2.94 | 2.01 | -1.13 | 7.11 | 1.86 | 1.81 | -1.72 | 5.28 | 4.52 | 2.18 | .16 | 8.63 |
| Child age | .78 | 1.38 | -1.48 | 3.87 | .92 | 1.24 | -.88 | 3.97 | .57 | 1.25 | -1.45 | 3.55 | 0.61 | 1.54 | -2.58 | 3.48 |
| Child male gender | 5.27 | 1.77 | 1.96 | 9.01 | 4.48 | 1.70 | .90 | 7.88 | 4.82 | 1.63 | 1.43 | 7.79 | 5.83 | 1.87 | 2.65 | 9.99 |
| Parent education | -.15 | .27 | -.76 | .36 | -.07 | .24 | -.58 | .36 | .18 | .20 | -.27 | .54 | -0.25 | .31 | -.92 | .34 |
| ECE Yrs experience | -.21 | .17 | -.54 | .16 | -.14 | .17 | -.47 | .22 | -.32 | .14 | -.60 | -.02 | -0.07 | .19 | -.40 | .35 |
| ECE Income | -.58 | .34 | -1.29 | -.01 | -.74 | .33 | -1.42 | -.19 | -.33 | .32 | -1.08 | .21 | -0.55 | .38 | -1.30 | .14 |
| ECE Workplace stressors | -.18 | .10 | -.39 | -.01 | -.21 | .09 | -.40 | -.04 | -.26 | .10 | -.48 | -.09 | -0.13 | .11 | -.37 | .07 |
| ECE DERS | -.11 | .06 | -.21 | .02 | -.09 | .06 | -.19 | .03 | -.10 | .06 | -.21 | .02 | -0.09 | .07 | -.22 | .05 |
| ECE Psychological distress | .13 | .06 | .03 | .27 | .18 | .05 | .10 | .30 | .12 | .06 | .02 | .24 | 0.09 | .07 | -.04 | .23 |
| ECE ACES | .10 | .64 | -1.11 | 1.42 | -.15 | .55 | -1.19 | 1.10 | .09 | .55 | -.81 | 1.34 | -0.08 | .70 | -1.33 | 1.45 |
|  |  |  |  |  |  |  |  |  |  |  |  |  |  |  |  |  |
| *Random effects* |  |  |  |  |  |  |  |  |  |  |  |  |  |  |  |  |
| Residual | 93.96 | 12.10 |  |  | 79.49 | 11.30 |  |  | 79.92 | 9.91 |  |  | 105.20 | 14.47 |  |  |
| ECE | 14.46 | 13.65 |  |  | 18.53 | 11.75 |  |  | 11.83 | 12.47 |  |  | 16.06 | 15.41 |  |  |
|  |  |  |  |  |  |  |  |  |  |  |  |  |  |  |  |  |
| -2 Restricted Log Likelihood | 1024.11 |  |  |  | 1007.78 |  |  |  | 1003.17 | 1003.17 |  |  | 1038.38 |  |  |  |
| AIC | 1028.11 |  |  |  | 1011.78 |  |  |  | 1007.17 | 1007.17 |  |  | 1042.38 |  |  |  |
| Pseudo-R^2^ | .11 |  |  |  | .13 |  |  |  | .12 |  |  |  | .10 |  |  |  |

Note: Bolded confidence intervals represent significant effects in bootstrapped analyses; ** *p* < .01, **p* < .05 in non-bootstrapped models. BRIEF-P: Behavior Rating Inventory of Executive Function- Preschool, Yrs: Years, ECE: Early childhood educator, AIC: Akaike’s Information Criterion.

**
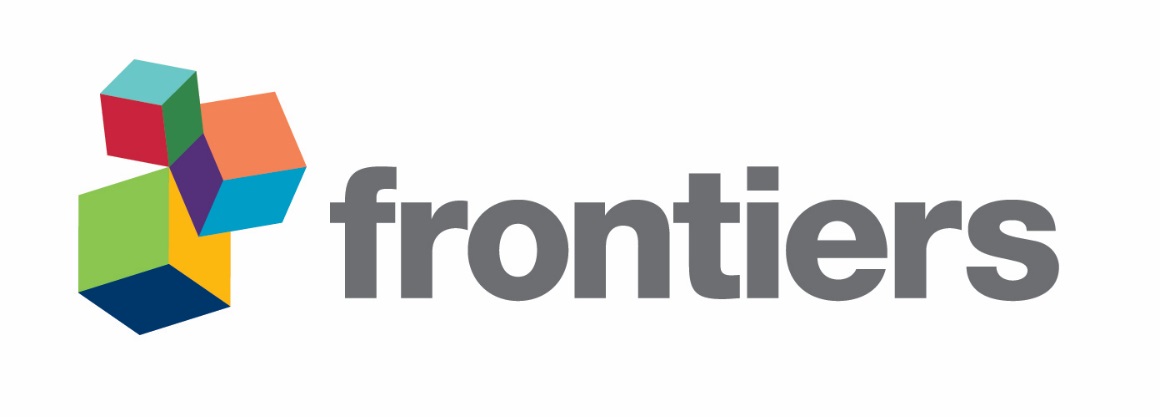
**

.
